# Morin inhibits *Listeria monocytogenes* virulence in vivo and in vitro by targeting listeriolysin O and inflammation

**DOI:** 10.1186/s12866-020-01807-6

**Published:** 2020-05-12

**Authors:** Gen Li, Guizhen Wang, Meng Li, Li Li, Hongtao Liu, Meiyang Sun, Zhongmei Wen

**Affiliations:** 1 Department of Respiratory Medicine, The First Hospital of Jilin University, Jilin University, Changchun, Jilin, 130021 China; 2 Key Laboratory of Zoonosis, Ministry of Education, Institute of Zoonosis, College of Veterinary Medicine, Jilin University, Changchun, 130062 China; 3 College of Food Engineering, Jilin Engineering Normal University, Changchun, China; 4 Department of Breast Surgery, Jilin Provincial Cancer Hospital, Changchun, China

**Keywords:** *Listeria monocytogenes*, Listeriolysin O,morin, Inflammation, Anti-infection

## Abstract

**Background:**

*Listeria monocytogenes* (*L. monocytogenes*) is a global opportunistic intracellular pathogen that can cause many infections, including meningitis and abortion in humans and animals; thus, *L. monocytogenes* poses a great threat to public safety and the development of the aquaculture industry. The isolation rate of *Listeria monocytogenes* in fishery products has always been high. And the pore-forming toxin listeriolysin O (LLO) is one of the most important virulence factors of *L. monocytogenes*. LLO can promote cytosolic bacterial proliferation and help the pathogen evade attacks from the host immune system. In addition, *L. monocytogenes* infection can trigger a series of severe inflammatory reactions.

**Results:**

Here, we further confirmed that morin lacking anti-*Listeria* activity could inhibit LLO oligomerization. We also found that morin can effectively alleviate the inflammation induced by *Listeria* in vivo and in vitro and exerted an obvious protective effect on infected cells and mice.

**Conclusions:**

Morin does not possess anti-*Listeria* activity, neither does it interfere with secretion of LLO. However, morin inhibits oligomerisation of LLO and morin does reduce the inflammation caused during *Listeria* infection.

## Background

*Listeria monocytogenes* (*L. monocytogenes*) is a ubiquitous gram-positive intracellular pathogen that frequently grows under the conditions used for food preservation and is regarded as the etiologic agent of a series of severe diseases, including meningitis, sepsis, and even life-threatening infections, in both humans and animals. *L. monocytogenes* poses a particularly serious threat to immunocompromised individuals, such as the elderly and pregnant women [1, 2]. The ingestion of food contaminated with *L. monocytogenes* is the main method of infection, and thus, this pathogen poses a significant threat and severe challenge to the food chain and the food production industry [3]. A high mortality rate is an important manifestation of *L. monocytogenes* infections, and it has been reported that nearly 19% of annual deaths induced by food borne infections are caused by *L. monocytogenes.* In fact, in an outbreak in 2011, hundreds of people were infected, and dozens died from the infection [4, 5], which suggests that *L. monocytogenes* is an important public health concern.

The successful establishment of infection is an important step in the process through which pathogenic bacteria evade the host immune system, and a series of complex virulence factors are crucial weapons carried by bacteria to exert virulence at each stage of the infection [6]. Multiple virulence factors are secreted during the intracellular lifecycle of *L. monocytogenes* to achieve colonization and infection in the host. Listeriolysin O (LLO), which is encoded by the *hly* gene, is one of the most important virulence factors of *L. monocytogenes*; this factor plays a central role in the process of bacterial escape from the phagosome [7] and belongs to the cholesterol-dependent cytolysin (CDC) family, which includes suilysin [8] and pneumolysin [9]. The pore-forming toxin is also a crucial factor involved in the cell-to-cell spread of *L. monocytogenes*; the virulence and pathogenicity of *L. monocytogenes* in which *hly* is mutated to inhibit its hemolytic activity were almost lost in mouse infection models [10, 11]. In addition, LLO can induce a variety of apoptosis and cytotoxicity pathways [12–14], which suggests that LLO is an important and effective drug target for the treatment of *L. monocytogenes* infection.

*L. monocytogenes* infection can induce a variety of inflammatory reactions, including encephalitis, osteomyelitis, andperitonitis [15–17], and activates the assembly of AIM2, NLRC4, and NLRP3 inflammasomes, which are multi-protein complexes that induce activation of the proinflammatory cysteine protease caspase-1 during the disease course as well as the secretion of important pro-inflammatory cytokines, such as IL-1β, IL-6, and TNF-α [18–20]. These excessive inflammatory reactions cause great harm to the body, particularly immunocom promised populations [21, 22]. The severe inflammation induced by *L. monocytogenes* is also a crucial part of the disease process. Therefore, effective alleviation of the inflammatory response might be an important strategy for the treatment of *L. monocytogenes* infection.

The natural ingredient morin, which can be found in various fruits and vegetables, is a safe, edible flavonoid with multiple biological activities, including the induction of apoptosis, the killing of tumor cells to fight cancer, and antioxidant activities [23–25]. In addition, morin inhibits the LPS-induced inflammatory response [26], and molecular simulations in our previous study showed that morin can effectively inhibit the pore-forming activity of purified LLO by directly binding to the protein at very low doses [27], which suggesting that morin is a potential drug precursor, particularly in the fight against *L. monocytogenes* infection. However, previous studies have only remained at the level of molecular mimicry, and no in-depth studies have been performed. Thus, further in vivo and in vitro studies are still needed to confirm the effect of morin in the treatment of *L. monocytogenes* infection and to provide a preliminary theoretical basis for further drug development of the compound.

## Results

### Morin has no influence on *L. monocytogenes* growth

**Fig. 1:**
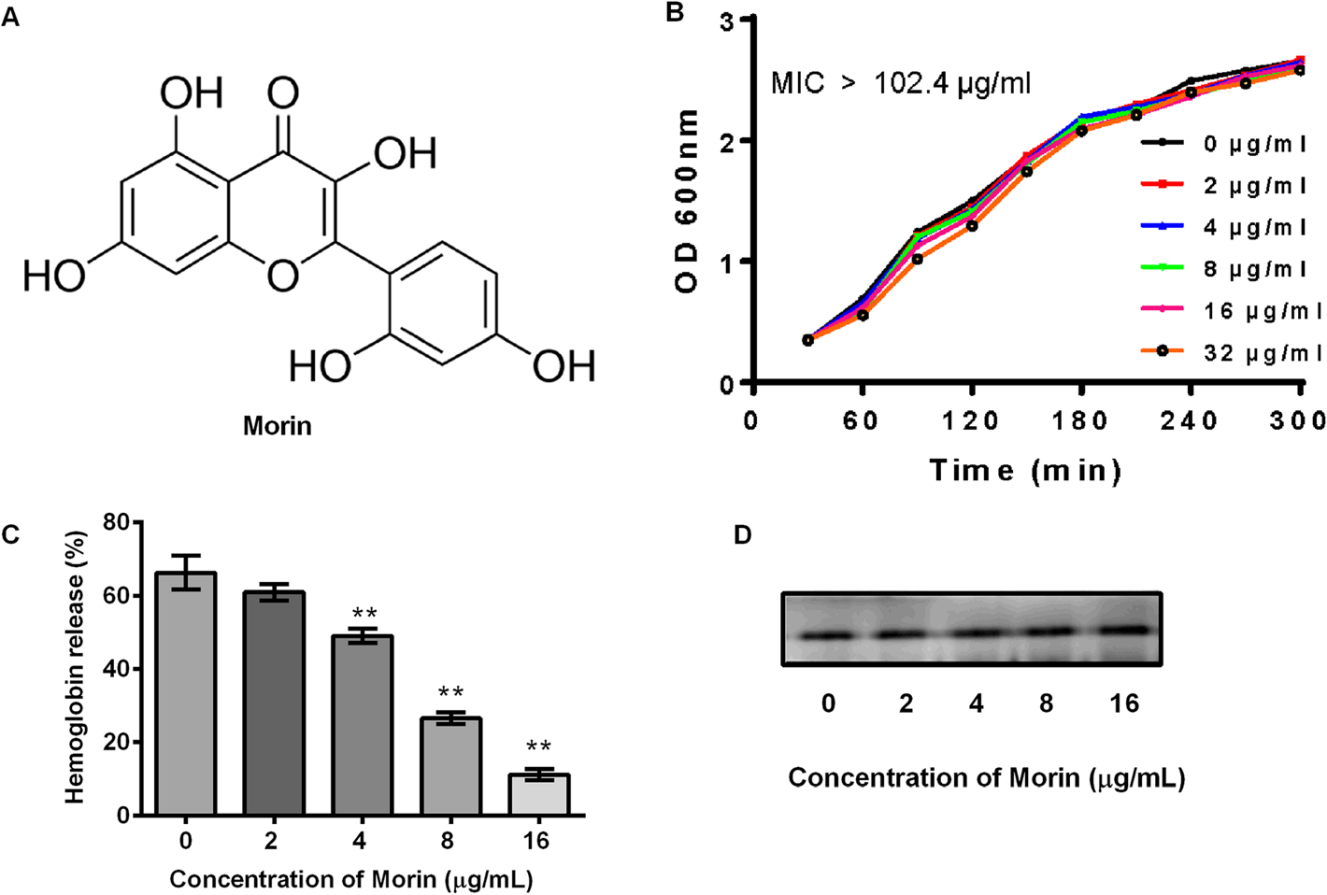
Inhibition of *L. monocytogenes* culture by morin. **a** Chemical structure of morin. **b** Growth kinetics curve of L95 cells treated with different concentrations of morin. L95 cells were incubated in TSB in the presence of various dosages of morin, and the growth of each sample was monitored every 30 min by measuring the value of OD_600 nm_ at each indicated time point. **c** Suppression of the hemolytic activity of L95 culture supernatants by morin. L95 cells were co-cultured with morin, and the co-cultured supernatants were harvested by centrifugation. The hemolytic activity was assessed by a hemolysis assay. The hemolysis percent of each co-cultured supernatant was compared with that of the positive control group (treatment with Triton X-100). **d** The expression of LLO in *L. monocytogenes* cultures treated with different concentrations of morin was assessed by Western blotting using a specific antibody against LLO. Three independent trials were performed for each index. * indicates *P* < 0.05, and ** indicates *P* < 0.01. (Student’s t-test)

Morin (Fig. 1a) is a kind of natural flavonoids and we found that the minimum inhibitory concentration of morin against L95 cells was more than 102.4 μg/mL. Furthermore, the addition of morin at various concentrations, including at the effective concentration (32 μg/mL) at which it functions, induced no obvious difference in the growth of L95 cells (Fig. 1b). Moreover, morin (16 μg/mL) clearly suppressed the function of lysing red blood cells in the bacterial culture (Fig. 1c), which is consistent with our previous finding that morin can directly bind to LLO in vitro [27]. However, in addition to direct binding, reducing the expression of LLO in the supernatant of the bacterial culture might also weaken the hemolytic activity. Therefore, we further tested the expression of LLO in the supernatant of bacterial cultures treated with different concentrations of morin and found that morin treatment did not interfere with the secretion of LLO (Fig. 1d). In summary, morin does not affect the growth of *Listeria*, which suggests that the use of morin for the treatment of *Listeria* infection would not impose too much pressure on the survival of the bacteria.

### Morin inhibits the oligomerization of LLO

**Fig. 2:**
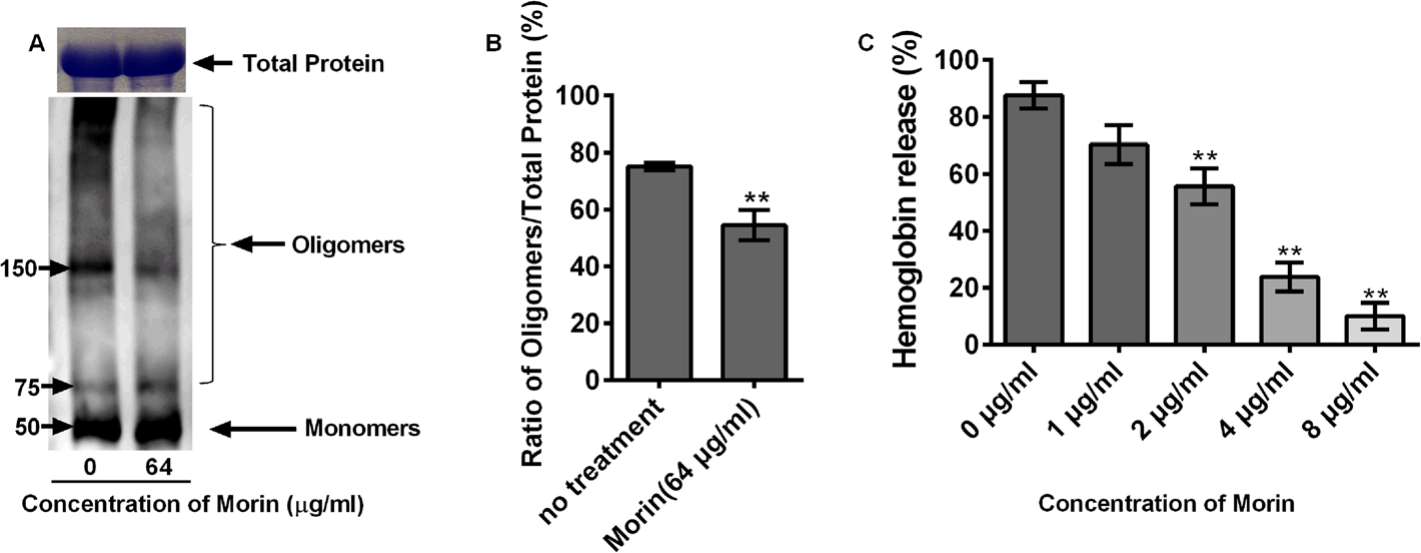
Inhibitory effects of morin on oligomer formation and the hemolytic activity of LLO. **a** and **b** Purified LLO proteins were treated with morin, and the inhibition of LLO oligomerization by morin was assessed by Western blotting. The total protein level in the groups without oligomerization induction was determined by coomassie blue staining. **c** Purified LLO protein was co-incubated with morin in PBS, and the co-cultured systems were harvested by centrifugation. The hemolytic activity was assessed by a hemolysis assay. Three independent trials were performed for each index.* indicates *P* < 0.05, and ** indicates *P* < 0.01. (Student’s t-test)

Our previous studies have shown that morin can bind directly to LLO to inhibit its hemolytic activity [27]. Here, we further demonstrated that the binding of morin and LLO effectively inhibited the formation of LLO oligomers and did not induce changes in the total protein in the various groups (Fig. 2a and b). The purified LLO protein was induced to oligomerize in vitro. The addition of morin into the reaction system caused the oligomeric bands to become narrow, significantly decreased the number of oligomers and reduced the hemolytic activity of LLO (Fig. 2c).

### Morin inhibits the secretion of inflammatorymediators in cells cultured with *L. monocytogenes*

**Fig. 3:**
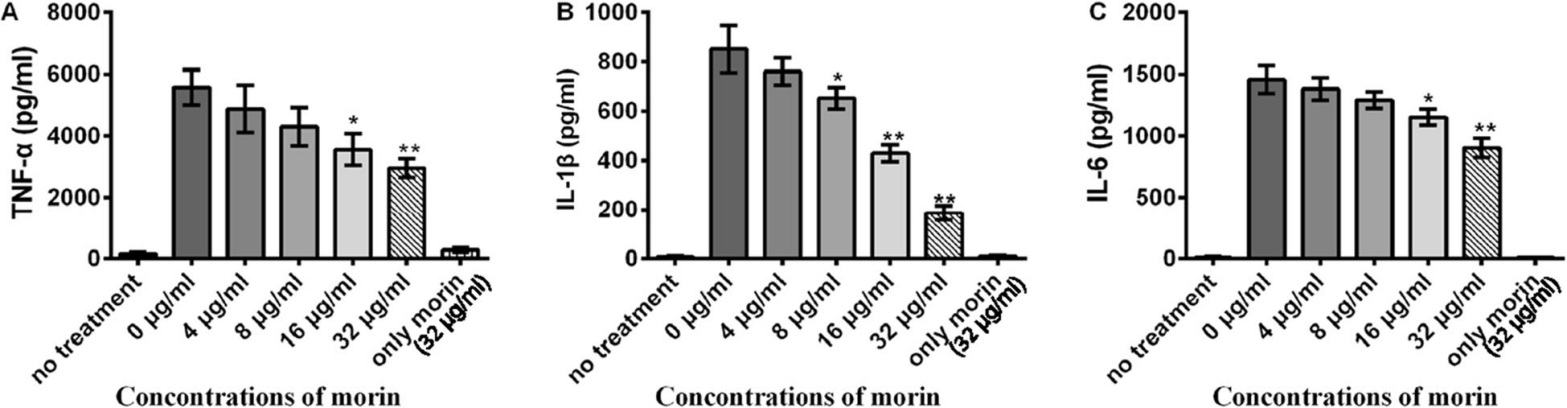
Inhibition of the production of inflammatory cytokines by morin in cells infected with *L. monocytogenes.* J774 macrophage-like cells were co-cultured with *L. monocytogenes* in the presence or absence of morin (0, 8, 16, and 32 μg/mL) at an MOI of 10 for 5 h. The co-culture supernatants were harvested by centrifugation, and the levels of TNF-α (**a**), IL-1β (**b**) and IL-6 (**c**) were detected by ELISA. Cells treated with morin in the absence of *L. monocytogenes* were used as a control. Three independent trials were performed for each index.* indicates P < 0.05, and ** indicates P < 0.01. (Student’s t-test)

All of the above results suggest that morin might play an inhibitory role in *Listeria* infection; therefore, we further studied the function of morin in *Listeria* infection of J774 cells. The secretion of TNF-α, IL-1β, and IL-6, which are considered important inflammatory mediators, was markedly down-regulated, which indicates that morin exerts a significant anti-inflammatory effect in vitro. In addition, the use of morin (32 μg/mL) alone exerted little effect on the expression of inflammatory factors compared with that observed with untreated cells (Fig. 3a-c).

### Morin can effectively alleviate the damage to cells infected with *L. monocytogenes*

**Fig. 4:**
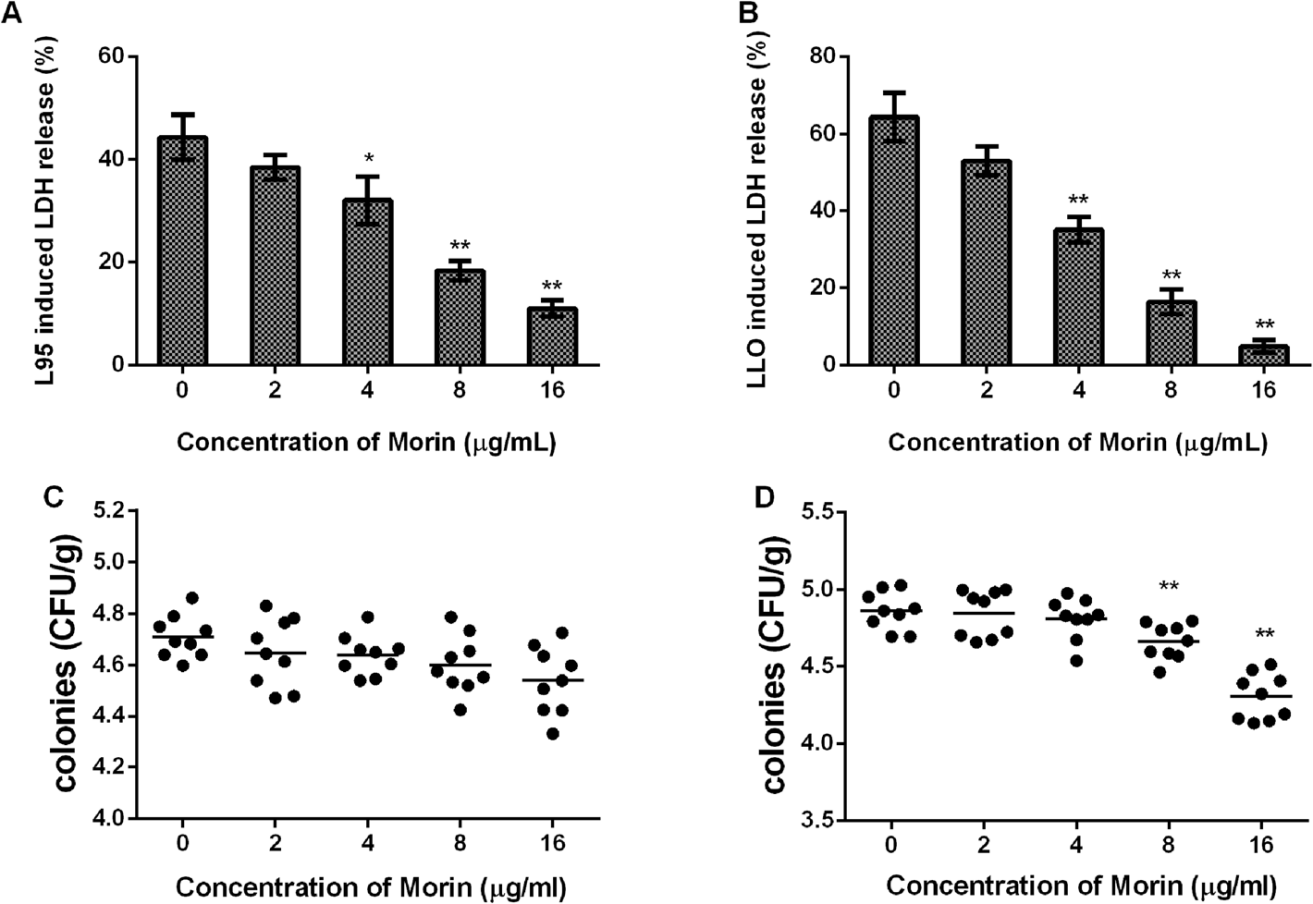
Morin-mediated attenuation of cell damage after infection with L95 cells or treatment with LLO*.* The level of LDH released into the supernatants of cells infected with L95 cells (**a**) or treated with LLO (**b**) in the presence or absence of various concentrations of morin was detected using a cytotoxicity detection kit. The extracellular (**c**) and intracellular (**d**) colonies were calculated by colonization. Three independent trials were performed for each index. * indicates P < 0.05, and ** indicates P < 0.01. (Student’s t-test)

To further explore the role of morin in *Listeria* infection in vitro, we detected the LDH levels in the cell culture supernatant of J774cellscocultured with L95 cells and different concentrations of morin, and treatment with morin also effectively reduced the release of LDH by the infected cells. In addition, morin itself exerted no cytotoxic effects on cells (Fig. 4a). In line with the results of bacterial infection, morin also significantly protected against LLO-induced cell damage (Fig. 4b), which further suggested that morin can play an anti-infective role by inhibiting LLO activity. Consistent with the results of the above-described studies, morin reduced the survival of intracellular bacteria but hardly affected extracellular bacteria (Fig. 4c and d). These results strongly suggest that morin exerts a significant protective effect during *Listeria* infection in vitro.

### Morin protects mice infected with *L. monocytogenes* and reduces the bacterial burden and inflammation in vivo

**Fig. 5:**
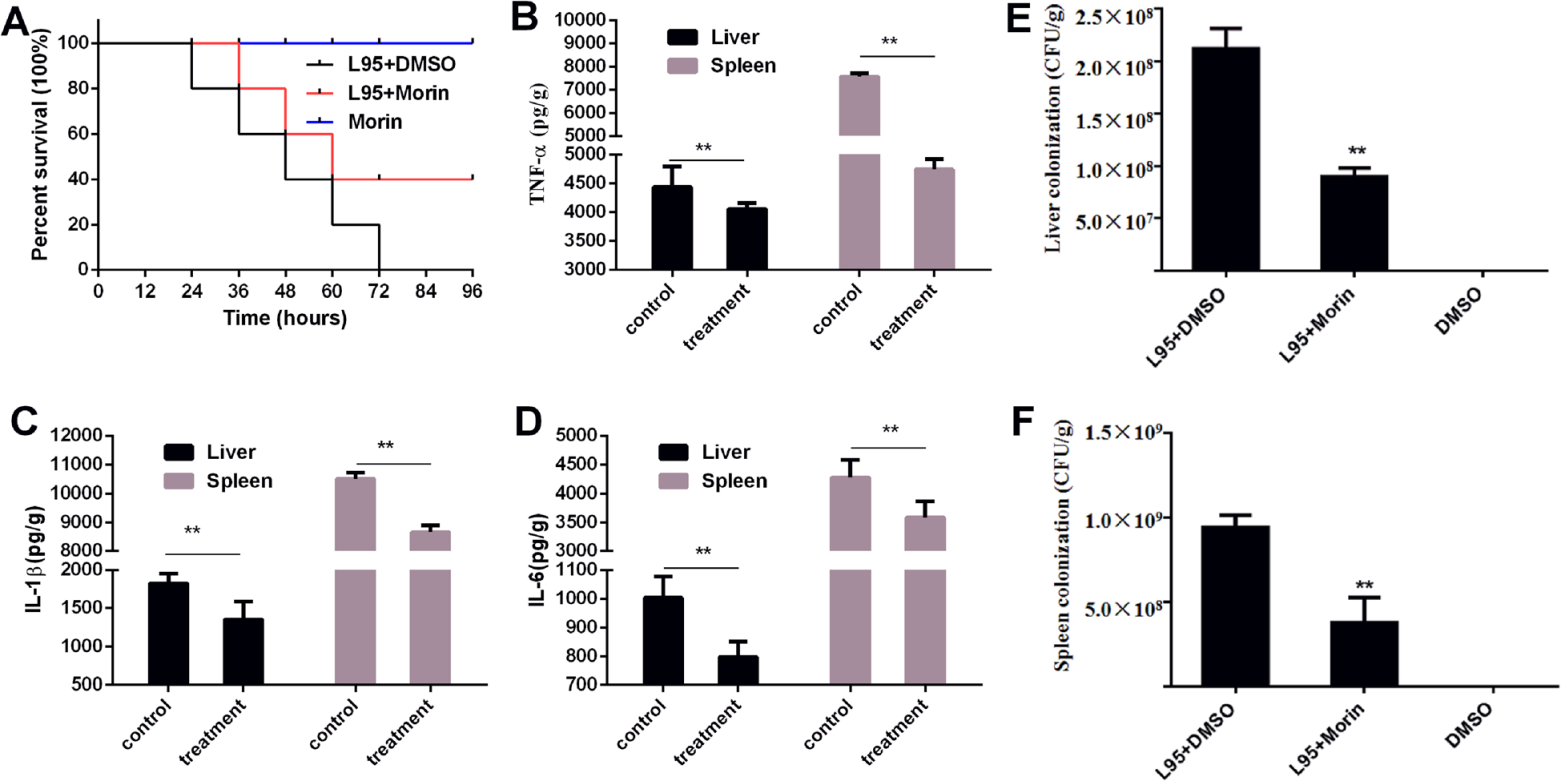
Protective effect of morin against *L. monocytogenes* infection in vivo. **a** Mice were intraperitoneally injected with 1 × 10^7^ CFUs of L95 cells and treated with 100 mg/kg morin or DMSO 2 h after infection and at 8-h intervals. The survival rate of the infected mice was observed for 96 h. The mice were intraperitoneally injected with 2 × 10^6^ CFUs of L95 cells and treated with 100 mg/kg morin or DMSO 2 h after infection and again at 8-h intervals. Forty-eight hours after infection, all of the mice were euthanized, the tissues of the liver and spleen were homogenized, and the number of colonies in ground tissue was calculated viaserial dilution (**e**-**f**). The levels of IL-6, IL-1β and TNF-α were detected by ELISA (**b**-**d**). Ten mice were arranged to each group for survival assays and three independent trials were performed (30 in total). For the inflammation and burden of the bacteria assays three mice were arranged to each group and three independent trials were performed (9 in total).* indicates *P* < 0.05, and ** indicates *P* < 0.01. (Student’s t-test)

To assess the therapeutic effect of morin in *Listeria* infections in vivo, we established a mouse *Listeri*a infection model. Mice were injected with 1 × 10^7^ CFU of L95 cells in the peritoneal cavity to observe the survival rate and 2 × 10^6^ CFU of L95 cells to determine the colonization of organs and inflammation of tissue. Mice died significantly after *Listeria* infection, but after subcutaneous administration of morin, the survival rate after infection with a high concentration of L95 cells was significantly increased compared with the control, indicating that morin has a protective effect on *Listeria* infection (Fig. 5a).

The in vivo effect of morin was further assessed by analyzing the bacterial burden in and detecting the inflammation of tissue from mice infected with L95 cells. A mouse model of *Listeria* infection was successfully established, and colonization in the tissues (liver and spleen) was significantly decreased by morin treatment (Fig. 5e and f). In accordance with the above-described results, as shown in Fig. 5b, c, and d treatment with morin also significantly relieved inflammation in the infected mice. Taken together, these data suggest that morin treatment can protect mice during in vivo *Listeria* infections.

## Discussion

*L. monocytogenes* is an important opportunistic food borne bacterium of both humans and animals and is the etiological agent of *listeriosis* and a series of fatal infections [28]. At present, the treatment of diseases related to *Listeria* infection in clinical practice mainly involves the application of antibiotics for the control or killing of pathogenic microorganisms [29]. However, an increasing number of *Listeria* strains that can survive antibiotic treatment are being discovered clinically at higher frequencies, and the problem of *Listeria* resistance is becoming more serious [30]. Over the past few decades, antibiotics have been used as the major treatment for nearly all types of bacterial infectious diseases in both humans and animals. However, the abuse, abnormal use or overuse of antibiotics has led to a series of adverse effects, particularly the acceleration of multidrug resistance in bacteria, and have caused more serious and alarming infections, which have resulted in some bacterial infections for which there are no available drugs [31]. Even more concerning, the development of new types of bacteriostatic drugs is occurring at a markedly slower rate compared with the generation of drug resistance, which has resulted in the current embarrassing and dangerous situation characterized by higher rates of bacterial resistance and pathogenicity and the availability weapons that can be used to fight against bacterial infections [32]. In recent decades, the development of a new anti-virulence strategy for the treatment of anti-bacterial infection has provided an effective and promising method for coping with the declining efficacy of traditional antibiotics [33]. Anti-virulence strategies differ from traditional antibiotics in terms of their antibacterial effects: anti-virulence strategies could involve inhibiting core elements in the bacterial lifecycle, exerting anti-infective activity and reducing bacterial pathogenicity by acting on virulence factors, and these effects would apply a relatively low survival pressure in comparison with antibiotics and are not conducive to the induction of bacterial drug resistance [32, 33]. LLO is a crucial virulence factor in the pathogenesis of *Listeria*, and the deletion of LLO can significantly inhibit bacterial escape from the phagosome and reduce the pathogenicity of the bacteria [10, 11], which suggests that LLO deletion could be regarded as an important and effective target in the therapy of *Listeria* infections. In addition, the severe inflammatory response caused by *Listeria* infection also causes harm to the infected host and particularly induces a risk for abortion in pregnant women [34], which indicates that attenuating the inflammatory response could bean important strategy for the treatment of *Listeria* infection.

At present, morin is widely used in the treatment of diseases. Only a small amount of orally administered morin can cross the blood-brain barrier to the brain, where as morin hydrate-loaded micellar nanocarriers show a markedly improved ability to cross the blood-brain barrier and play a therapeutic role in Alzheimer’s disease [35, 36], which suggests that the appropriate modification of the form of morin administered can improve its therapeutic effect on meningitis induced by *Listeria* infection. In addition, some studies have shown that morin also exerts potential therapeutic effects in the treatment of *Staphylococcus aureus* and *Streptococcus suis* infections [37, 38], which suggests that morin has potential as a broad-spectrum agent for the treatment of bacterial infections. Current studies have shown that LLO is associated with inflammation induced by *Listeria* infection, but the exact role remains unclear [39–41]. Therefore, whether there is a correlation between the anti-inflammatory activity of morin and its activity of inhibiting LLO is an interesting research direction in the future.

In our previous research, we found that morin can clearly suppress the hemolytic function of LLO, and molecular simulations demonstrated that the inhibitory effect was achieved by direct binding between morin and LLO to form an LLO-morin complex. The predicted binding site is the region of residues 420–470 in LLO, which is important for the interaction of morin with LLO, and the C1-C2 double-bond of morin (Fig. 1a) is also essential for this process [27]. In this study, we further investigated the potential protective function of morin in the treatment of *Listeria* infection and found that morin did not interfere with bacterial growth or expression of LLO protein. These findings further confirmed that the morin-mediated suppression of LLO hemolytic function is achieved by its direct action on the protein itself, which indicates that morin could be regarded as an anti-virulence agent and therapeutic candidate for *Listeria* infection. In addition, we also discovered that morin can significantly alleviate the secretion of inflammatory mediators induced by *Listeriain vitro* and in tissue cultures from the liver and spleen in vivo. The expression of the important proinflammatory cytokines TNF-α, IL-6, and IL-1β was reduced, and morin also exhibited effective protection against *Listeria* at the cell level in vitro and decreased the bacterial burden in vivo*.* The inflammatory response is a key defensive response of the host to exogenous pathogens. However, excessive inflammatory reactions induced by bacterial infection can also cause damage to the host. And our research found that morin can effectively lower the up-regulation of inflammatory factors produced by *Listeria* infection, indicating that morin can inhibit the inflammatory response induced by *Listeria* infection. These also suggest that we can further investigate whether morin will plays a role in *Listeria* infection-induced inflammatory diseases such as meningitis. In addition, a recent study found that morin could inhibit the formation of *Listeria* biofilms [42]. However, it is interesting that a cyclic dipeptide cyclo (L-leucyl- L-prolyl) can inhibit the hemolytic activity of *Listeria* and also reduce the formation of *Listeria* biofilms [43]. Whether the morin and cyclo (L-leucyl- L-prolyl) are similar in action is an important research direction in the future, and whether LLO is related to the biofilm formation process is also an interesting question worth thinking about.

All of these findings lay theoretical foundations for further study of morin as a candidate for the challenge of *L. monocytogenes* infection. Morin can be easily obtained in large quantities from a variety of plants and is suitable for large-scale production, which provides a powerful advantage for potential drug development. However, our work has only completed some preliminary theoretical research, and a series of in-depth studies are still needed for the development of morin as a new drug that can be clinically applied for the treatment of *L. monocytogenes* infection. In particular, further studies should identify a suitable form for administration and perform effective structural modifications to further enhance the effect of the drug.

## Conclusions

In this study, we demonstrated that morin exhibited no antibacterial activity against *L. monocytogenes* and did not influence the expression of LLO. However, morin significantly reduced the inflammation induced by *L. monocytogenes* infection in vitro and in vivo, alleviated cellular injury, protected infected mice and reduced the bacterial burden in vivo. These findings suggest that morin could be an effective and promising candidate to fight against *L. monocytogenes* infection.

## Methods

### Bacterial strains and reagents

*L. monocytogenes* strain ATCC 19115 (L95) was cultured in trypticase soy broth (TSB) with various concentrations of morin (purity ≥98%) that was purchased from Dalian Meilun Biotech Co., Ltd.

### Anti-*L.monocytogenes* activity testing

The minimum inhibitory concentration (MIC) of morin for L95 cells was evaluated as described in a previous study [14]. In brief, L95 cells were cultured to the logarithmic growth stage in TSB, and the density was adjusted to 1× 10^8^ CFUs/mL. Morin was diluted continuously with TSB medium, and the bacterial culture was added to 5× 10^5^ CFUs/mL. The growth of bacteria in the presence of different concentrations of morin was observed every 12 h for 48 h. For the determination of bacterial growth, the absorbance at OD_600 nm_ of L95 cells cultured in TSB with the indicated concentrations of morin at 37 °C was measured every 30 min for 5 h. Three independent trials were performed.

### Hemolysis assay

L95 cells were cultured in TSB with various concentrations of morin (0, 2, 4, 8, and16 μg/mL) at 37 °C for 5 h. Following centrifugation (10,000 rpm, 2 min), 100 μL of the supernatant from each co-culture sample was incubated with sheep erythrocytes (25 μL) and PBS (875 μL) for 20 min. The system was centrifuged, and the absorption value at OD_543nm_ of the supernatant was detected to determine the hemolytic activity; specifically, the activity of each sample was compared with that of the control sample (sheep erythrocytes treated with PBS supplemented with 2% Triton X-100), which was set as 100%. Three independent trials were performed.

The purified LLO protein was incubated with morin (0, 1, 2, 4, and 8 μg/mL), and the hemolytic activity in these samples was measured as described above. Three independent trials were performed.

### Western blotting assay

L95 cells were cultured in TSB with morin at 37 °C for 5 h. Following centrifugation, the supernatants of each sample were treated with Laemmli sample buffer, boiled for 10 min and separated on SDS-PAGE gels. After electrophoretic transfer, the LLO on the polyvinylidene fluoride membrane was blocked at room temperature for more than 2 h, and the polyvinylidene fluoride membrane was incubated with a primary rabbit anti-LLO antibody (Abcam, 1:2000) (original concentration 0.12 mg/ml) for 2 hand then with a corresponding secondary antibody (Proteintech, 1:3000) (original concentration 0.5 mg/ml) for 1 h. ECL detection reagents were used to visualize the signals on the PVDF membranes with a Tanon-4200 imager. Three independent trials were performed.

The purification of LLO protein and the induction of its oligomerization were performed as previously described [44]. In brief, LLO was incubated with morin (64 μg/mL) for 20 min, and oligomerization was induced under high salt conditions in vitro. First, LLO protein was incubated with morin for 30 min under acidic PBS (pH 5.5) conditions, and saturated potassium chloride was then added to a concentration of 0.3 mg/mL. The mixture was incubated for another 10 min, and sheep erythrocytes were then added to the reaction system. After treatment on ice for 5 min, the reaction samples were boiled with SDS-PAGE loading buffer lacking 2-hydroxy-1-ethanethiol. Oligomerization was detected by Western blotting as described above. Three independent trials were performed.

### Cell-line infections

L95 cells were cultured to the mid-logarithmicphase of growth (OD_600 nm_ of approximately 1.0), and the cells were washed with sterile PBS and then re-suspended in complete DMEM (HyClone) containing no fetal bovine serum (FBS).

J774 macrophage-like cells were grown in high-glucose DMEM at 5% CO_2,_ and the cells were plated in Corning 96-well plates (approximately2 × 10^4^ cells per well) and cultured for 16 h. The J774 cells were then co-cultured with L95 cells at 37 °C for 5 h at an MOI (multiplicity of infection) of 8 or treated with LLO protein (12 ng per well) that was pre-incubated with morin (0, 2, 4, 8, and16μg/mL) for 30 min at 37 °C for the detection of lactate dehydrogenase. The co-culture supernatant was diluted continuously, and the extracellular bacteria were determined by colony counting. For the assessment of intracellular bacteria, the supernatant of the culture medium was removed and washed three times with PBS, and gentamicin (20 μg/mL) was added to kill the residual extracellular bacteria. The cells were then lysed with 0.2% saponins (sigma), and the colony count was determined by continuous dilution. Three independent trials were performed.

J774 cells grown in Corning six-well plates (1 × 10^6^ cells per well) were infected with L95 cells in the presence of morin at an MOI of 10 at 37 °C for 4 h for inflammation assays. Three independent trials were performed.

### Inflammation assays

Following centrifugation (1000 rpm, 10 min), the inflammatory mediators (IL-1β, IL-6, and TNF-α) contained in each co-infection sample were determined using IL-1β, IL-6, and TNF-α ELISA kit (BioLegend, San Diego, CA, USA). Three independent trials were performed.

### Cytotoxicity assays

Following centrifugation (1000 rpm, 10 min), the lactate dehydrogenase level in the infected cell samples was assessed using a cytotoxicity detection kit (Roche, Basel, Switzerland). Samples treated with Triton or DMEM were used as controls. Three independent trials were performed.

### Animal experiments

Female BALB/c mice (6–8 weeks) were purchased from the Experimental Animal Center of Jilin University, and the animal experiments were approved by Jilin University (the First Hospital) institutional animal care committee and performed in accordance with their guidelines. All experimental animals were given sufficient food and water, provided sufficient ventilation, activity space and a good living environment. After the experiment, cervical dislocation was performed for euthanasia, and the experimental animals were not given pain during the whole process.

L95 cells were cultured to the mid-logarithmic growth stage (OD_600_ = 0.8–1.0), centrifuged, collected, washed three times with sterilized PBS and resuspended to a density of 1 × 10^8^ CFU/mL. The mice that were intraperitoneally injected with 1 × 10^7^ CFUs were administered subcutaneously 100 μg/g morin or DMSO (25 μL) 2 h after infection and then at 12-h intervals to assess the protective effect of morin. The mortality rate of the infected mice was observed for 96 h. In addition, mice intraperitoneally injected with the L95 cell suspension (2 × 10^6^ CFUs/mouse) were used for detection of the bacterial burden and inflammation in vivo, and morin was administered subcutaneously (100 mg/kg) 2 h after infection and then at 12-h intervals. DMSO was administered every 12 h in control animals. The mice were sacrifice by euthanasia 48 h after infection. The liver and spleen were homogenate processing in sterile PBS and cultured on TSB agar medium by spreading plate, then calculate the number of bacteria. The pro-inflammatory cytokines in the tissue supernatant collected after centrifuge were determined by ELISA. Ten mice were arranged to each group for survival assays and three independent trials were performed (30 in total). For the inflammation and burden of the bacteria assays three mice were arranged to each group and three independent trials were performed (9 in total) (Table 1).

**Table 1:** The details of animal assays arrangement

Test category	Survival			Colony counting			Inflammatory factors		
Group	infection	treatment	blank	infection	treatment	blank	infection	treatment	blank
**Number**	10/10/10	10/10/10	10/10/10	3/3/3	3/3/3	3/3/3	3/3/3	3/3/3	3/3/3
**Pepetition**	3								
**Infection routine**	intraperitoneal injection								
**Treatment**	subcutaneous injection								
**Time begin**	2 h after infection								
**Time stop**	96 h			48 h					
**Interval**	12 h								
**Details**	6–8 weeks, weight around of 20 g, female, get diet and water freely, euthanasia								

### Statistical analysis

All the experimental data are presented as the means ± SDs and were obtained from experiments that were repeated independently at least three times. The statistical analysis was conducted using GraphPad Prism 5.0. Differences with *P* values < 0.05 and P values < 0.01 are marked in the figures (Student’s t-test).

## Data Availability

The datasets used in the current study is available from the corresponding author upon reasonable request.

## Abbreviations

*L. monocytogenes*: *Listeria monocytogenes*
LLO: listeriolysin O
CDC: Cholesterol-dependent cytolysin
TSB: Trypticase soy broth
FBS: Fetal bovine serum
IL-1β: Interleukin-1 beta
IL-6: Interleukin-6
TNF-α: Tumor Necrosis Factor-α
PVDF: Polyvinylidene fluoride membrane
MOI: Multiplicity of infection
